# Improving laboratory quality and capacity through leadership and management training: Lessons from Zambia 2016–2018

**DOI:** 10.4102/ajlm.v10i1.1225

**Published:** 2021-04-30

**Authors:** Felicity Gopolang, Fales Zulu-Mwamba, Davy Nsama, Annika Kruuner, Dailes Nsofwa, Ishmael Kasvosve, Royce Gomo, Tiny Motlhabane, Bhavna Chohan, Olusegun Soge, Daniel Osterhage, Nancy Campbell, Michael Noble, Ann Downer, Jean-Frederic Flandin, Anya Nartker, Catherine Koehn, Linda K. Nonde, Aaron Shibemba, Clement B. Ndongmo, Martin Steinau, Lucy A. Perrone

**Affiliations:** 1 Department of Global Health, Schools of Public Health and Medicine, International Training and Education Center for Health (I-TECH), University of Washington, Seattle, Washington, United States; 2 Laboratory Services Unit, Directorate of Clinical Care and Diagnostic Services, Ministry of Health Zambia, Lusaka, Zambia; 3 ZAMBART, Lusaka, Zambia; 4 Laboratory Quality Management Systems, Centers for Disease Control and Prevention (CDC) Zambia, Lusaka, Zambia; 5 Faculty of Health Sciences, University of Botswana, Gaborone, Botswana; 6 ImmunoGene Labs, Ruwa, Zimbabwe; 7 Medical Laboratory Technology Department, Institute of Health Sciences, Gaborone, Botswana; 8 Department of Pathology and Laboratory Medicine, University of British Columbia, Vancouver, British Columbia, Canada; 9 HIV and AIDS Twinning Center Program, American International Health Alliance (AIHA), Lusaka, Zambia; 10 Center for Disease Control and Prevention (CDC) Zambia, Lusaka, Zambia

**Keywords:** leadership, quality management, workforce development

## Abstract

**Background:**

Competent leadership and management are imperative for delivering quality laboratory services; however, few laboratory managers receive job-specific training in organisational management and leadership.

**Objective:**

To develop and evaluate participants’ competencies in organisational leadership and management as measured through learner and laboratory quality improvement assessments.

**Methods:**

This professional development programme employed a mentored, blended learning approach, utilising in-person didactic and online training, with the practical application of a capstone project in the laboratories. Programme impact was evaluated through a series of pre- and post-laboartory assessments using the Stepwise Laboratory Improvement Process Towards Accreditation checklist, as well as learner-competency assessments through online quizzes and discussions.

**Results:**

From 2016 to 2018, 31 managers and quality officers from 16 individual laboratories graduated from the programme having completed capstone projects addressing areas in the entire laboratory testing process. Laboratories increased their compliance with the International Organization for Standardization 15189 standard and all but two laboratories significantly increased their accreditation scores. Two laboratories gained three stars, two laboratories gained two stars, and five laboratories gained one star. Five laboratories subsequently achieved International Organization for Standardization 15189 accreditation in 2019.

**Conclusion:**

This programme taught leadership theory to laboratory managers and allowed them to implement leadership and management practices in the laboratory setting. Programmes such as this complement existing laboratory quality management training programmes such as Strengthening Laboratory Management Toward Accreditation.

## Introduction

Medical laboratories are a critical component of healthcare because they provide essential data for effective patient care, pathogen detection, disease surveillance and response. Enabling access to quality laboratory services is a challenge in low-resource settings^1^ and many laboratories in resource-constrained countries provide poor quality diagnostic testing with incorrect, unreliable, or significantly delayed test results. Competent laboratory management and leadership are vital for delivering quality laboratory services and laboratories need leaders who can utilise their resources effectively in a variable healthcare environment.^2^ These leadership skills are required to effect beneficial change in complex healthcare settings^3^ and work effectively across disciplines; however, they are not skills that laboratory managers (LMs) commonly cultivate during conventional academic programmes.^4^ Few laboratory supervisors ever receive formal laboratory management and leadership training for their roles.^5,6,7^ Effective laboratory quality management requires that laboratory supervisors have not only the technical knowledge of quality management systems (QMS) and national and international standards for medical laboratory quality such as the International Organization for Standardization (ISO)15189 but also the strong leadership and managerial skills to lead their staff and drive accreditation efforts.^8,9,10,11,12^

The Strengthening Laboratory Management Toward Accreditation (SLMTA) programme was launched in 2009,^13,14^ and provides iterative quality management training to hundreds of laboratory personnel. SLMTA programme addresses common workforce knowledge gaps in resource-constrained settings via a multi-workshop implementation model. The guide for the Stepwise Laboratory Improvement Process Towards Accreditation^15^ checklist was endorsed by the World Health Organization Regional Office for Africa in 2011. It serves as a benchmarking tool to monitor laboratory conformity to the ISO15189 quality standard and the SLMTA programme. As of 2019, SLMTA has been implemented in 1368 laboratories globally and of these 191 (7.16%)^16^ have been ISO15189 accredited. While significant progress has been made in laboratory quality through QMS training programmes such as SLMTA in the last decade,^17^ strengthening the impact of these programmes across the continent and increasing representation of the laboratory sector in the upper levels of healthcare governance requires further investments in leadership and management training for LMs and directors.^18^

Public health leadership training is evidently beneficial to clinical practitioners and policymakers^19,20^ and there is a need for wider access to similar programmes for laboratory professionals.^21^ However, there are limited formal leadership programmes available.^22,23,24,25,26,27^ To address this gap, the Certificate Program in Laboratory Leadership and Management^28^ was developed in 2013 at the University of Washington in consultation with global laboratory practice experts. The goal of the Certificate Program is to build a scalable professional development programme aimed at building the leadership and management skills of laboratory staff in supervisory positions. The participant criteria ultimately ensured that participants were in the leadership position to make substantive and impactful improvements in their laboratory’s testing quality and operations. The programme was implemented in Zambia for two years starting in 2016 to strengthen leadership and management competencies of LMs and quality assurance officers from key tertiary public and military hospital laboratories. Also, this programme aimed to improve the laboratories’ quality of diagnostic services and their compliance with the Stepwise Laboratory Improvement Process Towards Accreditation (SLIPTA) checklist towards achieving ISO15189 accreditation. We aim to describe the effectiveness of this laboratory leadership programme in two Zambian cohorts, using the laboratories’ compliance with the SLIPTA checklist as the main outcome measure.

## Methods

### Ethical considerations

Approval to conduct the study was received from the Human Research Ethics Committee, University of New England (approval number HE13-240).

### Program design and implementation

**FIGURE 1:**
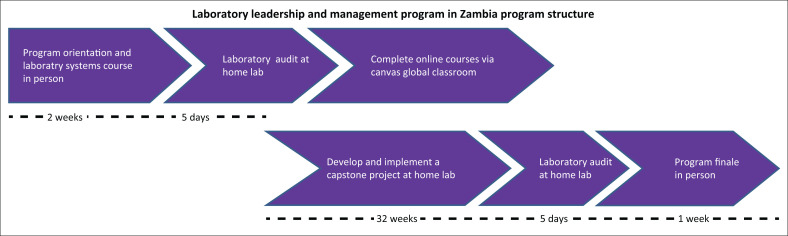
Structural overview for the laboratory leadership and management programme in Zambia, 2016–2018. Two cohorts of participants from 16 laboratories across Zambia participated from 2016–2018, each cohort taking 9 months to complete the programme work. Both programme years utilised a similar approach to adult experiential learning, utilising a blended solution of online and face-to-face instruction, a robust online discussion board as well as close faculty and mentorship support for individual capstone projects conducted at participant’s home laboratories. Orientation and finale sessions were conducted in Lusaka. Seventeen laboratory managers from 16 laboratories completed the 2016–2017 programme and completed 16 unique capstone projects. For the second cohort, 16 laboratory managers and 15 quality managers completed the programme and conducted 15 unique capstone projects.

The Certificate Program was implemented in two cohorts from 2016–2018; each programme cohort completed course and project works in 9 months. This culturally appropriate and effective^25^ programme employed a mentored, competency-based,^29^ blended learning approach. It was designed for adult learners and courses were delivered in-person and online. Participants delivered a capstone project, which is an individualised, practical application of a quality improvement (QI) project (Figure 1). In each cohort, two in-person sessions bookended the online coursework. The in-person sessions served as the programme orientation and finale sessions.

The orientation session introduced participants to the programme structure, content, learning goals and expectations, mentor-participant guidelines, the online learning management system (LMS; Canvas™ Learning Management System, London),^30^ and the laboratory assessment and audit tools to be used (e.g. SLIPTA).^15^ Following the orientation, participants returned to their worksites where they discussed the programme with their staff and chief medical superintendent before starting the baseline audit process and online coursework. The results of the baseline audits identified the CP focus area and provided a guideline for the development of CP work plans.

The curriculum for the 2016 cohort included five courses from the University of Washington delivered sequentially, the first on *Laboratory quality and systems* (delivered in-person), followed by *Laboratory leadership, Laboratory management, Communicating laboratory information*, and *Implementing diagnostic technology*. The latter topics were delivered online via the Canvas LMS. Each online course was four weeks long and included 20 h – 25 h (~5–6 h/week) of mixed media instruction and a weekly discussion. Each course was followed by a 2–3-week instruction intermission during which participants submitted their CP-related assignments. The CP was a customisable QI project designed and implemented by participants at their laboratories with close support from mentors and faculty. The CP process began after the orientation session, with a baseline laboratory audit conducted over a period of 1 week using the SLIPTA checklist.^15^ Through their CPs, participants were to exemplify team leadership and improve teamwork through delegation and a system of accountability.

The curriculum for the second cohort (2017–2018) included a University of British Columbia quality assurance online curriculum for quality assurance officers. This online course was delivered in seven online modules via the Blackboard LMS system (Blackboard Inc., Washington, District of Columbia, United States) and conveyed traditional QMS principles of Shewhart,^31,32^ Deming,^33,34,35,36^ Crosby,^37^ and Juran^38,39^ with additional perspectives by faculty. Curriculum courses delivered instructions necessary for compliance with the ISO quality and competence (ISO15189) requirements and expectations for medical laboratories. The LMs in the second cohort undertook advanced training based on Kouzes and Posner’s textbook and workbook ‘The Leadership Challenge’.^40^ The LMs participated in the online coursework concurrently with the quality assurance officers and conducted joint CPs in their home laboratories.

Both programme cohorts ended with an in-person meeting where participants presented their CPs to their peers, mentors and faculty and received a programme completion certificate.

### Participant and mentor selection

The programme was specially designed for quality assurance officers and LMs who are currently working in a managerial role in a health laboratory; participants and mentors were selected by the programme’s selection committee following specific eligibility criteria.

Mentors had an average of 20 years’ experience in the clinical laboratory field and were paired with up to seven participants. Mentors provided on-site and remote coaching using various communication channels, including the LMS discussion board, email, Skype (Microsoft Corp., Redmond, Washington, United States), and WhatsApp™ (Facebook Inc., Menlo Park, California, United States) calls. Mentors provided step-by-step support and motivated participants to apply knowledge gained from the global classroom to address management challenges such as staff resistance to change, particularly from some long-serving staff members. Mentors also reinforced messages of individual leadership and accountability by encouraging laboratory staff at all service levels to implement smaller QI projects. The staff were to identify gaps related to the LM’s CP and led efforts to find solutions. Mentors also coached participants to organise and conduct meetings with the laboratory staff, the quality team, and the senior hospital administrators. These proposed meetings were aimed at engaging all laboratory staff and the hospital administration with the implementation and review of the laboratory improvement program.

### Learner and programme evaluation

The programme was evaluated based on both learner and facility impact. Learner outcome metrics included self-rated competency and graded assessments including graded participation in the weekly online discussion board accompanying each course, course exams and CP-related assignments (analysis of laboratory audit result, CP project proposal, work plan development, implementation update, final report and project presentation).^41^ Course surveys, exit interviews, and facility pre-programme and post-programme SLIPTA checklist audits conducted by the Ministry of Health were also used to evaluate the programme. At the end of each programme year, via an online programme survey, qualitative programme feedbacks were received from the participants on various aspects of the programme. Participants identified the most valued aspect of the programme and evaluated the curriculum quality, the CP process, and the mentor’s support. Also, a post-programme evaluation survey was conducted by an independent organisation in 2018.^42^ The survey utilised a Likert scale rating system to collect anonymised data from both cohorts on how participants felt their abilities had changed since they graduated from the programme. All quantitative and qualitative evaluation data were collected from survey responders and analysed using Excel software (Microsoft Corp., Redmond, Washington, United States).

## Results

### Demographics and graduation rate

Participants of both programmes were selected using established eligibility criteria from key laboratory facilities as indicated by the Zambia Ministry of Health (Table 1). Overall, 31 individuals completed the programme with 16/17 (94%) graduating in 2016 and 26/31 (84%) in 2018. These graduates (25 men and 6 women) conducted their programme work at 16 individual hospital laboratories from all nine provinces in Zambia (Table 2). Nine LMs completed both cohorts. Eight mentors from Zambia, Botswana, and Zimbabwe (three men, five women) supported each paired programme participant for an average of 3 h per week.

**TABLE 1:** Participant and mentor selection criteria, Zambia, 2016–2018.

Selection metric	Participant characteristics	Mentor characteristics
Current position level	Manager or supervisor in a clinical laboratory hospital or public health laboratory (early to mid-career)	Senior-level clinical or public health laboratory professional with experience in laboratory management
Education minimum	Diploma (bachelor’s or master’s degree preferred)	Graduate-level degree (medical doctor or Doctor of Philosophy preferred)
Job experience	5+ years of laboratory technical experience 1+ years of laboratory supervisory experience and currently supervising 3+ staff	10+ years of laboratory technical experience 5+ years of clinical or public health laboratory supervisory experience
Minimum skills required	Responsible for some aspect of laboratory operations and management Minimally experienced in supervising other laboratory staff Conversant with basic statistical methods	Experienced in managing a clinical or public health laboratory including the following: Direct supervision of staff Responsibility for the management of laboratory operations including procurement and budgets Interaction with clients such as government agencies and the public Basic knowledge of the local and international legal framework, laws and regulations governing laboratory practice Experienced in analysing and communicating laboratory data in reports or manuscripts; and experienced in publishing in peer-reviewed journals
Language skills	High proficiency in reading, writing and speaking English	High proficiency in reading, writing and speaking English
Computer skills	Comfortable, daily computer user; experienced using e-mail and the internet to access the programme’s online Learning Management System and search for related materials	Comfortable, daily computer user; experienced using e-mail and the internet to access the programme’s online Learning Management System
Career potential (participants)	Envisions a career with increasing responsibilities in clinical and public health laboratory management, and the potential for roles in laboratory and public health leadership	Willing and able to commit to: Being involved with participants during the entire 9 months of the programme Weekly check-in and coach participants (vacation and family emergency time excluded) Motivating, encouraging and challenging his or her participant to ensure they complete the programme Working alongside the participant on their capstone project
Programme commitment (mentors)	Desires to mentor others	-

**TABLE 2:** Programme demographics, Zambia, 2016–2018.

Programme year	Participants	Mentors
2016–2017	16 laboratories from nine provinces 17 laboratory managers	Eight mentors from Zambia, Botswana, and Zimbabwe
2017–2018	15 laboratories from nine provinces 16 laboratory managers (11 from cohort 1) 15 quality assurance officers	Four mentors from Zambia and Botswana
Total	31 participants (25 men and six women) from 16 individual clinical laboratories.	Eight mentors (three men and five women). Mentors had an average of 20 years’ experience in the clinical laboratory field

### Capstone project scope and success

Thirty-one CPs were completed by graduates in these two years and the CP topics addressed a range of issues on the total laboratory testing process (Table 3). In addition to these formal projects, supplemental QI projects were undertaken by other staff in the laboratory adjacent to the CP’s topical area. These supplementary projects which were undertaken by the general laboratory staff also contributed to the improved laboratory performance and addressed issues such as updating standard operating procedures to minimise specimen cross-contamination, implementing new duty rosters for daily equipment maintenance activities during public holidays and weekends and phlebotomy service task-shifting. The smaller QI projects strengthened both the internal and external laboratory communication channels and improved laboratory safety via the introduction of hand-washing facilities, controlled laboratory access, and routine Class II Biological Safety Cabinet smoke tests.

**TABLE 3:** Capstone project topic areas, Zambia, 2016–2018.†

Quality management subject area	Number of projects conducted	Examples of project progress‡
Management review	1	Management reviews are now being conducted
Corrective action	3	Improved follow-up of non-conformities to closure from 58% to 83% of events
Addressing pre-analytical causes of error	5	40% reduction in the incidence rate of pre-analytical errors; decrease in the specimen rejection rate
Customer satisfaction	2	Increase in the customer satisfaction rate from 67% to 81%
Analytic phase testing processes, laboratory efficiency	4	Improvement of turnaround time in GeneXpert testing by 44%
Equipment management	2	Creation of a comprehensive equipment management system service schedule and contracts
External quality control processes	1	Monitoring system developed for external quality assessment performance across all tests
Documents and records system	7	Quality manuals and standard operating procedures developed
Personnel management	1	Increase of 35% in the documentation of personnel details, development and implementation of an objective competency evaluation programme
Stock management	1	Development and utilisation of a stock card system
Post-analytical phase processes	4	Improvement of the quality of laboratory report for clinicians, development of new systems for report collection

† , 31 projects were completed at 16 laboratories between 2016–2018.

‡ , These are examples from individual laboratories.

### Quality improvement progress

**FIGURE 2:**
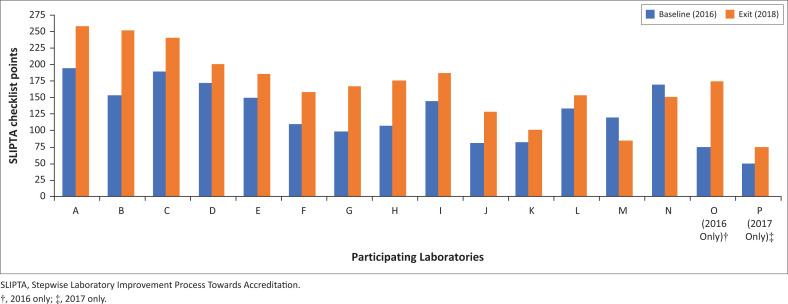
Changes in laboratory audit scores before (2016) and after (2018) the Laboratory leadership and management programme in Zambia, 2016-2018. Participants and representatives from the Ministry of Health conducted baseline Stepwise Laboratory Improvement Program Towards Accreditation audits of each laboratory at the beginning (2016) and end of the programme (2018). Audit scores are shown as whole numbers with a maximum score of 275 points.

The Ministry of Health conducted baseline (the beginning of each programme year) and exit (the end of the 9-month programme) SLIPTA audits. Both audits were utilised as benchmarking tools to measure the impact of the CP. After the programme period in 2018, the SLIPTA checklist audit scores of 14 out of the 16 participating laboratories (87.5%) increased (Figure 2), with nine laboratories also improving their SLIPTA star rating. Two laboratories gained three SLIPTA stars, another two gained two SLIPTA stars, and five gained one SLIPTA star. Of the seven other laboratories, six maintained their star rating while one laboratory lost a star rating. Three laboratories achieved five SLIPTA stars by the end of the programme and five of these participating laboratories have achieved ISO15189 accreditation^16^ at the time of this writing.

### Learner satisfaction

**FIGURE 3:**
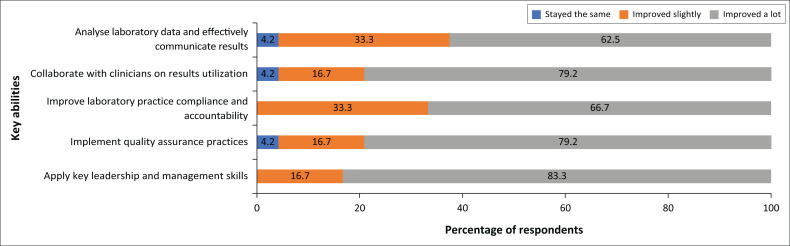
Participants’ self-perceived changes in key abilities after laboratory leadership and management programme in Zambia, 2016–2018. A Likert scale-based survey conducted of all programme graduates was conducted in 2018 by an external organisation. Graduates of the programme self-reported key changes in abilities as a result of the programme (*n* = 24 respondants) and percentage of each response were calculated and shown here.

Participants self-reported significant improvements of key competencies as a result of the programme as indicated by internal (Table 4) and external surveys (Figure 3). All participants reported improvement in their leadership and management knowledge and skills as well as laboratory practice compliance. More than 95% reported improved competencies in various other laboratory abilities such as critical analyses and interpretation of laboratory data, communication, collaboration with clinicians on result utilisation, improvement of laboratory practice compliance and accountability in line with national and international standards, implementation of essential quality assurance practices (timeliness, reliability and accuracy of testing), and application of leadership and management skills. Participants also involved other laboratory staff in learning by downloading recorded lectures for others to watch offline as a team and discussed weekly topics as a group. The programme was also highly rated by mentors as indicated from both internally and externally conducted surveys.

**TABLE 4:** Qualitative feedback from participants about the programme, Zambia, 2016–2018.

The most valued aspect of the programme	Feedback on the coursework	Feedback on the capstone project	On mentoring support
‘The [*entire*] process of identifying the project topic, identifying specific action points and measuring outcome and its significance to service delivery.’	‘I benefited a lot from the [*diagnostics*] course. Even principles I had heard of before were explained much more clearly and enhanced my understanding. I also liked the hands-on/real life assignment questions that helped me think through how we can overcome some of the challenges in laboratory management and leadership in our country.’	‘Many of the concepts learnt during the course were a core component of the project. The project was able to implement aspects of teamwork, conflict management, and listening skills just to mention a few.’	‘He helped me to align my thoughts and ideas better when developing the proposal. Through the initial discussions we had, I was able to come up with *[a]* proposal that had direction and clear purpose of what it is I wanted to achieve.’
‘The most important things I learnt from this project are working as a team in the laboratory. Everyone took their responsibilities very seriously given that they had to complete tasks within specified periods in this project. There was a sense of ownership and this was a delight to witness. Collaborating with other departments of the hospital was also very good. There was a lot to learn from other departments. I found all parts [of the program] to be valuable but the planning part and implementation process proved to be most valuable.’	‘This [*leadership*] course came at the right time in that it encompasses real issues in my laboratory. Issues like relationship building, how to build trust when working as a team. I am glad that I will use these skills.’	‘I had to have all stakeholders involved in finding a solution to the identified issue. I developed leadership, negotiation, problem-solving and communication skills.’	‘Mentors were very experienced and were above board. I salute them.’
‘I would like to take a lead in ensuring that QMS is implemented. This course has empowered me with knowledge and skills relevant to guide my colleagues. I will be more usable in the area of quality in our lab.’	‘This is a very well-organized [*leadership*] course. The knowledge I got far exceeded my expectations. I can definitely recommend this course to anyone who is serious about learning and/or improving their leadership and management skills.’	‘My project involved working with teams comprising members of varying qualifications and abilities. I had to apply leadership skills such as building strong teams in order to bring everyone together and find common solutions to problems we had identified in the baseline audit. Also, I had to apply skills learned on communicating laboratory information and encouraging/persuading my target audience to buy into the idea that I wanted us to pursue.’	‘She offered me technical support regarding implementation of the planned activities and how to put the information together in a progress report.’

QMS, quality management systems.

## Discussion

This programme set out to improve the leadership and quality management skills of a cohort of laboratory supervisors in Zambia including improving their competencies in management, communication, policy development, laboratory data analysis, and international quality management principles to improve the laboratories’ ability to deliver quality clinical and public health services. The blended learning programme was successful in achieving a > 80% graduation target rate for both cohorts. Participants indicated in surveys that the programme improved their leadership and management skills and subsequently their laboratory’s performance. All respondents reported that they thought the programme applied to their work and that they would recommend the programme to their peers. The continuous support and motivation from faculty and mentors^43,44,45^ ensured participants were supported during the entire programme period. Also, the employment of an effective and reliable online LMS to deliver high-quality asynchronous online courses and support a robust real-time discussion board to foster the cultivation of a strong community of practice among each cohort contributed to the high retention rate observed. The online discussion board was utilised daily for communication and enabled participants to share best laboratory leadership, management and advocacy practices with their peers and receive valuable feedback. Importantly, the programme was valued by participants because it delivered both theoretical and practical applications of effective laboratory leadership and management.

The CP was a unique component of the programme, unifying the entire laboratory around a common goal and fostering a strong working relationship between the management team and the technical staff. This process resonated the importance of strong teams in furthering an organisation’s mission. This blended learning programme is therefore unique in that the modular online curriculum is adaptable to any environment, allowing for customisation with location-specific needs and inclusion of a global audience and experts, regardless of the time zone. The potential for local ownership and expansion of this programme is immense as evidenced by the breadth of project topics participants undertook as well as the adjacent QI projects. The projects improved participant’s leadership and management skills as well as their laboratory’s QMS in line with ISO15189 (as measured by the pre- and post-programme SLIPTA audits). The projects also addressed internal indicators of laboratory quality such as specimen rejection rate, turnaround time and client satisfaction. All participating laboratories demonstrated QI; however, not all these improvements are captured by the SLIPTA audits. Notably, the structured programme content and sustained faculty and mentor engagement are implicated in these observed laboratory QMS improvements and contributed to the international recognition of three participating laboratories. As of the time of this writing, five of these participating laboratories have now achieved ISO15189 accreditation.

Some challenges were encountered in the two-and-a-half-year programme. Specifically, management staff changes in some facilities within the two programme periods challenged the continuum of QI. Particularly, staff turnover in 2017 correlated with lower QI in many of the participating laboratories. Also, other implementing partners at times were simultaneously present on-site with programme mentors which reduced available contact time with mentees. Mentors expressed challenges such as mentees not responding to communications and availing themselves during distant mentorship. Personal time management was the only participant self-reported challenge; concurrently meeting programme and work responsibilities was demanding for participants. As such, future efforts will be made by the programme developers to condense the programme length based on feedback, offer all of the coursework online to minimise on-site time, and include content pertaining to personnel time management and motivation particularly when there are competing interests.

Distance learning programmes that include significant components of field or work-based training are proving to be highly effective in fostering practical competency development and behaviour change in learners^46^ and the results of this programme for LMs is no exception. Importantly, the modular online curriculum and blended format of the programme is permissive of adoption and adaptation by local institutions such as universities and professional associations. Adoption and implementation by local organisations will have added benefit to laboratory professionals either by contributing to university degrees such as diplomas or continuing education credits as part of an annual licensure programme or career advancement points for leadership positions. Cost elements of the programme include faculty time, mentor honorarium, data plans for participants, LMS maintenance and logistical costs for on-site coaching and in-person meetings. Programme implementation costs could likely be reduced should the programme be converted to a completely online programme including mentorship. However, the impact and quality of an entirely online programme are yet to be evaluated. Although massive open online courses offer exciting opportunities to distribute knowledge on a massive and global scale, a full understanding of their effectiveness to deliver competency-based training to healthcare professionals remains limited and further research is warranted.^47^ The value of laboratory leadership programmes such as the one we describe here are starting to receive greater attention from the public health practice community^48^ and should be supported alongside other efforts to strengthen national laboratory systems.^49^

### Limitations

This programme was limited to a selected group of participants from Zambia who were selected based on their position in their organisation or their occupation. As such, success in this programme was dependent on staff continuity in the programme and vulnerable to disruptions caused by staff reassignment. Should the programme become more financially sustainable through user fees, the global audience could be expanded and no longer tied to priority facilities as determined by external donors.

### Recommendations

- Leadership and management training, such as this training programme, is highly recommended as it can lead to measurable impacts in the laboratory.
- Leadership and management training programmes such as this programme are highly recommended to complement existing QMS training programmes such as SLMTA.
- Professional development programmes for healthcare practitioners delivered through online learning platforms should also include an applied project where learned theory from the global classroom can be applied to the job.

### Conclusion

This programme affirms the impact of formal leadership and management training on laboratory capacity and builds on previous investments to improve quality, system operability, and preparedness. The programme emphasised the functional practices of organisational leadership and effectively supplemented quality management training programmes; it can be implemented alongside other efforts to strengthen national laboratory systems.

## References

[1] Martin R, Barnhart S. Global laboratory systems development: Needs and approaches. Infect Dis Clin North Am. 2011;25(3):677–691. 10.1016/j.idc.2011.05.001 21896367

[2] Olmsted SS, Moore M, Meili RC, et al. Strengthening laboratory systems in resource-limited settings. Am J Clin Pathol. 2010 9;134(3):374–380. 10.1309/AJCPDQOSB7QR5GLR 20716792

[3] Smith T, Stankunas M, Czabanowska K, De Jong N, O’Connor S, Fowler Davis S. Principles of all-inclusive public health: Developing a public health leadership curriculum. Public Health. 2015 2;129(2):182–184. 10.1016/j.puhe.2014.12.001 25707932

[4] Renner AS, Bennett S. Developing the next generation of laboratory leaders [homepage on the Internet]. Clinical Laboratory News 2015 11 01 [cited 2019 Jul 23]. Available from: https://www.aacc.org/publications/cln/articles/2015/november/developing-the-next-generation-of-laboratory-leaders

[5] DeBoy JM, Beck AJ, Boulton ML, Kim DH, Wichman MD, Luedtke PF. Core courses in public health laboratory science and practice: Findings from 2006 and 2011 surveys. Public Health Rep. 2013;128 Suppl 2(Suppl 2):105–114. 10.1177/00333549131280S215 23997310 PMC3730012

[6] Kasvosve I, Ledikwe JH, Phumaphi O, et al. Continuing professional development training needs of medical laboratory personnel in Botswana. Hum Resour Health. 2014 8;12:46. 10.1186/1478-4491-12-46 25134431 PMC4141587

[7] Report of the WHO laboratory leadership and management training programme meeting, Lyon, France, 12–13 May 2011. Lyon: World Health Organization; 2011.

[8] International Organization for Standardization (ISO). ISO 15189: 2012 medical laboratories – Requirements for quality and competence. Geneva: International Organization for Standardization; 2012.

[9] Albert H, De Dieu Iragena J, Kao K, Erni D, Mekonen T, Onyebujoh PC. Implementation of quality management systems and progress towards accreditation of national tuberculosis reference laboratories in Africa. Afr J Lab Med. 2017;6(2):a490. 10.4102/ajlm.v6i2.490 PMC5523922 28879161

[10] Andric LR, Massambu CG. One laboratory’s progress towards accreditation in Tanzania. Afr J Med. 2014;3(2), Art. #202, 4p. 10.4102/ajlm.v3i2.202 PMC5637807 29043185

[11] Mothabeng D, Maruta T, Lebina M, Lewis K, Wanyoike J, Mengstu Y. Strengthening laboratory management towards accreditation: The Lesotho experience. Afr J Lab Med. 2012;1(1), Art. #9, 7p. 10.4102/ajlm.v1i1.9 PMC5644518 29062729

[12] Viegas SO, Azam K, Madeira C, et al. Mozambique’s journey towards accreditation of the national tuberculosis reference laboratory. Afr J Lab Med. 2017;6(2):a491. 10.4102/ajlm.v6i2.491 PMC5523919 28879162

[13] Yao K, McKinney B, Murphy A, et al. Improving quality management systems of laboratories in developing countries: An innovative training approach to accelerate laboratory accreditation. Am J Clin Pathol. 2010;134(3):401–409. 10.1309/AJCPNBBL53FWUIQJ 20716796

[14] World Health Organization Regional Office for Africa. Strengthening laboratory management towards accreditation [homepage on the Internet]. [cited 2019 Jul 23]. Available from: https://slmta.org/

[15] World Health Organization Regional Office for Africa. WHO guide for the Stepwise Laboratory Improvement Process Towards Accreditation (SLIPTA) in the African Region [homepage on the Internet]. Version 2:2015. [cited 2019 Jul 23]. Available from: https://www.afro.who.int/sites/default/files/2017-06/slipta-checkist0711.pdf

[16] SLMTA.org. Laboratories that have achieved accreditation [homepage on the Internet]. [cited 2020 Jun 25]. Available from: https://slmta.org/accredited-labs/

[17] Nkengasong JN, Mbopi-Keou FX, Peeling RW, Yao K, Zeh CE, Schneidman M. Laboratory medicine in Africa since 2008: Then, now, and the future. Lancet Infect Dis. 2018 11;18(11):e362–e367. 10.1016/S1473-3099(18)30120-8 29980383 PMC13081755

[18] Alemnji GA, Zeh C, Yao K, Fonjungo PN. Strengthening national health laboratories in sub-Saharan Africa: A decade of remarkable progress. Trop Med Int Health. 2014 4;19(4):450–458. 10.1111/tmi.12269 24506521 PMC4826025

[19] Doherty J, Gilson L, Shung-King M. Achievements and challenges in developing health leadership in South Africa: The experience of the Oliver Tambo Fellowship Programme 2008–2014. Health Policy Plan. 2018;33(suppl_2):ii50–ii64. 10.1093/heapol/czx155 30053036 PMC6037070

[20] Nakanjako D, Namagala E, Semeere A, et al. Global health leadership training in resource-limited settings: A collaborative approach by academic institutions and local health care programs in Uganda. Hum Resour Health. 2015;13:87. 10.1186/s12960-015-0087-2 26581196 PMC4650924

[21] Best M, Sakande J. Practical recommendations for strengthening national and regional laboratory networks in Africa in the Global Health Security era. Afr J Lab Med. 2016;5(3), a471. 10.4102/ajlm.v5i3.471 PMC5433810 28879137

[22] Laboratory leadership curriculum [homepage on the Internet]. Chicago, IL: Clinical Laboratory Management Association; 2015 [cited 2019 Jul 23]. Available from: http://www.clma.org/p/cm/ld/fid=18

[23] Lab management university [homepage on the Internet]. Chicago, IL: American Society for Clinical Pathology; 2015 [cited 2019 Jul 23]. Available from: http://www.ascp.org/lmu

[24] Laboratory leadership service (LLS) [homepage on the Internet]. Atlanta, GA: Centers for Disease Control and Prevention; 2015 [cited 2019 Jul 23]. Available from: https://www.cdc.gov/lls/index.html

[25] Perrone LA, Confer D, Scott E, et al. Implementation of a mentored professional development programme in laboratory leadership and management in the Middle East and North Africa. East Mediterr Health J. 2016;22(11):832–839. 10.26719/2016.22.11.832 28177114

[26] Clinical laboratory leadership and management certificate program [homepage on the Internet]. Washington, DC: American Association of Clinical Chemistry. [cited 2019 Jul 23]. Available from: https://www.aacc.org/education-and-career/online-certificate-programs/certificate-programs/clinical-laboratory-leadership-and-management-certificate-program

[27] Emerging leader program [homepage on the Internet]. Washington, DC: Association of Public Health Laboratories. [cited 2019 Jul 23]. Available from: https://www.aphl.org/professional_development/Pages/ELP.aspx

[28] Certificate program in laboratory leadership and management [homepage on the Internet]. University of Washington, I-TECH. [cited 2019 Jul 23]. Available from: https://edgh.washington.edu/project/lab-certificate

[29] Ned-Sykes R, Johnson C, Ridderhof JC, Perlman E, Pollock A, DeBoy JM. Competency guidelines for public health laboratory professionals: CDC and the Association of Public Health Laboratories. Morb Mortal Wkly Rep. 2015;64(1):1–81. 25974716

[30] Instructure. Canvas Learning Management System. Salt Lake City, UT: Instructure Corporation; 2016. [cited 2019 Jul 23]. Available from: http://www.canvaslms.com

[31] Shewhart WA. Economic control of quality of manufactured product/50th anniversary commemorative issue. Milwaukee, WI: American Society for Quality; 1980.

[32] Shewhart WA. Statistical method from the viewpoint of quality control. Washington. The Graduate School, The Department of Agriculture.; 1939.

[33] Deming WE. The essential deming: Leadership principles from the father of quality. New York, NY: McGraw-Hill; 2012 12 11.

[34] Deming.org. The fourteen points for management [homepage on the Internet]. [cited 2020 Jun 20]. Available from: https://www.deming.org/theman/theories/fourteenpoints

[35] Deming WE. Out of the crisis. Cambridge, MA: The Massachusetts Institute of Technology (MIT) Press; 2018.

[36] Deming WE. Quality, productivity and competitive position. Cambridge, MA: Massachusetts Institute of Technology; 1982.

[37] Crosby PB. Quality is free. The art of making quality certain. New York, NY: McGraw-Hill; 1979.

[38] Juran J, Godfrey A. Juran’s quality handbook. 5th ed. New York, NY: McGraw-Hill; 2000.

[39] Juran JM. Managerial breakthrough: A new concept of the manager’s job. New York, NY: McGraw-Hill Companies; 1964.

[40] Kouzes JM, Posher BZ. The leadership challenge: How to make extraordinary things happen in organizations. 6th ed. Somerset: John Wiley& Sons Inc.; 2017.

[41] Kirkpatrick DL, Kirkpatrick JD. Evaluating training programs: The four levels. 3rd ed. San Francisco, CA: Berrett-Koehler; 2006.

[42] Cardia Services. [cited 2019 Jul 23]. Available from: http://www.cardeaservices.org/

[43] Kapanka AR. Journey to the millennium: Mentoring in the clinical laboratory. MLO Med Lab Obs. 1998 5;30(5):44–46. 10179686

[44] Beck SJ, Laudicina RJ. Passing the torch: Mentoring the next generation of laboratory professionals. Clin Lab Sci. 2001;14(1):33–36. 15633492

[45] Laudicina RJ. Mentoring for retention and advancement in the multigenerational clinical laboratory. Clin Lab Sci. 2001;14(1):48–52. 15633495

[46] Joynes, C. Distance learning for health: What works a global review of accredited post-qualification training programmes for health workers in low and middle-income countries [homepage on the Internet]. [cited 2020 Jun 20]. London International Development Centre (LDIC); 2011. Available from: https://lidc.ac.uk/_assets/DL4H%20Main%20Report.pdf

[47] Rowe M, Osadnik CR, Pritchard S, Maloney S. These may not be the courses you are seeking: A systematic review of open online courses in health professions education. BMC Med Educ. 2019 9 14;19(1):356. 10.1186/s12909-019-1774-9 31521150 PMC6744630

[48] Albetkova A, Isadore J, Ridderhof J, et al. Critical gaps in laboratory leadership to meet global health security goals. Bull World Health Organ. 2017;95(8):547–547A. 10.2471/BLT.17.195883 28804163 PMC5537757

[49] Opio A, Wafula W, Amone J, Kajumbula H, Nkengasong JN. Country leadership and policy are critical factors for implementing laboratory accreditation in developing countries: A study on Uganda. Am J Clin Pathol. 2010;134(3):381–387. 10.1309/AJCP6KMOTCLISGJ3 20716793

